# Fengbaisan suppresses endoplasmic reticulum stress by up-regulating SIRT1 expression to protect rats with chronic obstructive pulmonary diseases

**DOI:** 10.1080/13880209.2020.1806335

**Published:** 2020-09-08

**Authors:** Yu Wang, Nan-xiang Su, San-gai Pan, Xiao-ping Ge, Xing-ping Dai

**Affiliations:** a Institute of Integrative Chinese Medicine, Xiangya Hospital, Central South University, Changsha, Hunan, China; b Department of Traditional Chinese Medicine, The Second Xiangya Hospital, Central South University, Changsha, Hunan, China; c Emergency Department, Tianjin Emergency Center, Tianjin Changsha, China; d Department of Geriatrics, Changsha Ninth Hospital, Hunan, China

**Keywords:** TIMP-1/MMP-9, apoptosis, airway inflammation

## Abstract

**Context:**

Our previous study found that Fengbaisan improved chronic obstructive pulmonary diseases (COPD).

**Objective:**

To elucidate the mechanism of Fengbaisan in COPD.

**Materials and methods:**

Rats in Model, FBS, FBS + DMSO and FBS + EX527 groups received cigarette smoke extract (CSE) inhalation and intratracheal instillation of lipopolysaccharide to establish COPD model. Normal group received room air and normal saline. The COPD rats were given Fengbaisan (1 mL/d) or combined with EX527 (5 mg/kg/2 d) by intraperitoneal injection. Human lung carcinoma (A549) cells were treated with 10% CSE, 10% serum-containing Fengbaisan or EX527. We observed lung percentage of forced expiratory volume in first 0.3 sec to forced vital capacity (FEV0.3/FVC), inspiratory resistance (RI) and lung dynamic compliance (Cdyn) of rats. The lung pathological changes, the number of inflammatory cells and neutrophils, inflammatory factor, apoptosis, gene and protein expression were examined.

**Results:**

SIRT1 was downregulated in lung tissues of COPD rats and CSE-induced A549 cells. Fengbaisan enhanced FEV0.3/FVC (74.28%) and Cdyn (0.28 cm H_2_O/mL/s), and reduced RI (0.48 mL/cm H_2_O) of COPD rats. Moreover, Fengbaisan promoted SIRT1 expression, and repressed TIMP-1/MMP-9 expression. Fengbaisan enhanced apoptosis and the expression of GRP78, caspase-12 and caspase-3. The inflammatory factor levels, the number of inflammatory cells and neutrophils, and lung lesions were inhibited by Fengbaisan in COPD rats. The influence conferred by Fengbaisan was abolished by EX527.

**Discussion and conclusions:**

Fengbaisan inhibits endoplasmic reticulum stress and inflammation reaction by up-regulating SIRT1 expression to improve COPD. Therefore, Fengbaisan may be an effective Chinese medicine for treating COPD.

## Introduction

Chronic obstructive pulmonary disease (COPD) is a common chronic respiratory disease characterized by persistent airflow limitation. The pathological changes of COPD are chronic bronchitis and emphysema, which can further develop into pulmonary heart disease and respiratory failure (Baliatsas et al. 2017). COPD leads to a significant decrease in labour capacity and quality of life of COPD patients, and increases the risk of death (Minai et al. 2010; Wrobel et al. 2012). Standard treatment has little effect, whereas the drug resistance and toxic side effects are obvious. Therefore, these drugs are not suitable for long-term use.

Chinese medicine may play a greater role in the treatment of COPD. In the past two decades, our team has sought to determine the effect of Fengbaisan on the treatment of COPD. Fengbaisan is a Chinese medicine, mainly composed of *Astragalus membranaceus* Schischkin (Fabaceae), *Panax ginseng* C.A. Mey (Araliaceae), *Lilium brownii* F.E.Br. ex Miellez (Liliaceae), *Polygonatum sibiricum* Redouté (Asparagaceae), *Dioscorea oppositifolia* L. (Dioscoreaceae), *Ophiopogon japonicus* (Linn. f.) Ker-Gawl. (Asparagaceae), *Aster tataricus* L. f. (Asteraceae) and *Salvia miltiorrhiza* Bge. (Lamiaceae). Fengbaisan alleviates lung lesions in COPD rats, improves inflammatory cell infiltration and alveolar wall rupture, and reduces the enlargement of the alveolar space (Su et al. 2005). Fengbaisan represses the expression of MMP-9 and TIMP-1 in the bronchial and lung tissues of COPD rats and regulates the imbalance of MMP-9/TIMP-1 (Wang and Su 2011; Wang et al. 2014). Fengbaisan increases γ-GCS expression and SOD activity in the lung tissue of COPD rats, and it plays a vital role in anti-airway remodelling, antioxidative stress and delays the progression of COPD (Pan et al. 2009). Although the efficacy of Fengbaisan has been partially confirmed and affirmed, Chinese medicine has complex ingredients and exerts effects on the body through multiple components, targets and links. Thus, it is difficult to explore its mechanism, which is also one of the important reasons hindering the development of Chinese medicine.

Silent information regulator factor 2 related enzyme 1 (SIRT1), a protein widely present in human tissues and cells, attracts much attention in respiratory diseases. SIRT1 affects apoptosis through mechanisms such as oxidative stress, inflammation and endoplasmic reticulum stress (ERS), and then participates in the occurrence and development of many diseases. SIRT1 expression in the lung tissues of smoker and smoker with COPD is significantly reduced with respect to non-smokers, suggesting that SIRT1 plays an important protective role in lung tissue damage (Rajendrasozhan et al. 2008). SIRT1 protects lungs from cigarette smoke-induced oxidative stress by deacetylating FOXO3 (Yao et al. 2014). At the same time, SIRT1 regulates the ratio of TIMP-1/MMP-9 in the lungs of COPD patients to attenuate the occurrence and development of COPD and emphysema (Nakamaru et al. 2009; Yao et al. 2013; Trocme et al. 2015). Therefore, SIRT1 plays a crucial role in protecting lung cells and delaying cell death by regulating oxidative stress and inflammation (Hwang et al. 2013). And SIRT1 expression in lung cells can be used as a therapeutic target for COPD. Recent studies have shown that melatonin reduces apoptosis and inhibits NLRP3 inflammatory bodies by up-regulating the expression of SIRT1 to improve COPD (Peng et al. 2018; He et al. 2019b). Therefore, increasing the levels of SIRT1 in tissues may be a novel idea for COPD treatment.

Several studies have documented that many drugs in Fengbaisan have the effect of up-regulating SIRT1 expression and inhibiting ERS. For example, astragalus polysaccharides inhibit ochratoxin A-induced immune stress by activating AMPK-dependent SIRT1 *in vivo* and *in vitro* (Liu et al. 2018). *Salviae miltiorrhizae* exerts anti-inflammatory effects by regulating SIRT1/NF-κB signalling pathway (Liu et al. 2019). Licochalcone A exerts antioxidant and anti-inflammatory effects by up-regulating SIRT1 expression (Hou et al. 2019). Therefore, we established a COPD rat model to clarify whether Fengbaisan could inhibit ERS by up-regulating SIRT1 expression and improve COPD.

## Materials and methods

### Experimental animals

Sprague Dawley (SD) rats (male, aged 6-week-old, weighted 200–250 g) were obtained from Changsha Dongchuang Experimental Animal Science and Technology Service Center. All rats were raised in specific pathogen-free conditions and given free access to water and food. All protocols were authorized by the Ethics Committee of Xiangya Hospital.

### Rat model of COPD

The rat model of COPD was established by combining cigarette smoke with lipopolysaccharide (LPS) in accordance to the previous study (Wang et al. 2014). In short, rats were anaesthetized with 10% chloral hydrate and treated with LPS (200 μg/100 μL) by intratracheal instillation on the 1st and 14th day. Subsequently, the rats were exposed to cigarette smoke generated by Furong cigarettes (Hunan Tobacco Industrial Company, Hunan, China) (13 mg tar/cigarette) in a chamber (30 cm × 45 cm × 54 cm) for 30 min twice a day for 28 days except for the 1st and 15th day. Then, we judged whether the establishment of the rat model of COPD was successful by observing and testing the general condition and pulmonary function of rats. COPD rats exhibited symptoms and signs such as cough, shortness of breath, lethargic, slow movements, excessive sputum, anorexia, and yellow hair. Lung percentage of forced expiratory volume in first 0.3 s to forced vital capacity (FEV0.3/FVC), inspiratory resistance (RI) and lung dynamic compliance (Cdyn) of the rats was assessed using Forced Manoeuvres Pulmonary Function Testing (BUXCO, Wilmington, NC, USA). Compared with normal rats, COPD rats displayed a decrease of FEV0.3/FVC% and Cdyn, and an increase of RI.

### Composition and preparation of Fengbaisan

Fengbaisan was prepared as previously described (Wang et al. 2014). Briefly, total weight of whole drugs (122 g) was immersed in water for 30 min, fried on soft fire twice. Then, the two fried juices were merged, filtered with the membrane filter, and disinfected. Eventually the mass concentration of containing crude drug was 2.34 g/mL. Above all herbal components were purchased from the Pharmacy of the Second Xiangya Hospital of Central South University and identified by the Traditional Chinese Medicine Teaching and Researching Section, Xiangya Medical College, Central South University.

### Experimental protocol

Male SD rats were randomly assigned to five groups (n = 8 each): (1) Normal group; (2) Model group; (3) FBS group; (4) FBS + DMSO group; and (5) FBS + EX527 group. Normal group received room air, intratracheal instillation of normal saline; Model group received cigarette smoke, intratracheal instillation of LPS, intraperitoneal injection of normal saline; FBS group received cigarette smoke, intratracheal instillation of LPS, intraperitoneal injection of Fengbaisan (1 mL/d); FBS + DMSO group received cigarette smoke, intratracheal instillation of LPS, intraperitoneal injection of Fengbaisan and DMSO; FBS + EX527 group received cigarette smoke, intratracheal instillation of LPS, intraperitoneal injection of Fengbaisan and EX527 (5 mg/kg/2 d). Drug treatments were performed 1 h before cigarette smoke exposure or LPS instillation. Fengbaisan treatment was performed every day for 4 weeks. EX527 treatment was performed every two days for 4 weeks. On the next day after the experiment, the pulmonary function of rats was tested, including FEV0.3/FVC, Cdyn and RI. Then, all rats were fasted for 12 h and water deprivation for 4 h before anaesthesia. And rats were anaesthetized by intraperitoneal injection of 10% chloral hydrate (3 mL/kg). Lung tissues, bronchoalveolar lavage fluid (BALF), blank serum and different drug-contained serum were obtained from the rats of each group.

### Cell culture and treatment

Human lung carcinoma (A549) cells were obtained from public cell banks (ATCC, Manassas, VA, USA). The cells were cultured in RPMI-1640 medium (Sangon Biotech, Shianghai, China) supplemented with 1% penicillin/streptomycin. All the cells were incubated in a humidified atmosphere at 37 °C and 5% CO_2_.

### Cell model of COPD

A549 cells were treated with 10% CSE to induce COPD. We judged whether the establishment of the cell model of COPD was successful by detecting the apoptosis of A549 cells. COPD cells exhibited an increase of apoptosis with respect to normal A549 cells. Then, we collected serum from the rats in Model, FBS, FBS + DMSO and FBS + EX527 groups to treat A549 cells. A549 cells were randomly assigned to six groups: (1) Control (Ctrl) group: A549 cells cultured in serum-free RPMI-1640 medium; (2) Cigarette smoke extract (CSE) group: A549 cells were treated with 10% CSE. (3) Blank serum group: the CSE-treated A549 cells were treated with 10% blank serum; (4) FBS serum: the CSE-treated A549 cells were treated with 10% serum containing Fengbaisan; (5) FBS serum + DMSO: the CSE-treated A549 cells were treated with 10% serum containing Fengbaisan and DMSO; (6) FBS serum + EX527: the CSE-treated A549 cells were treated with 10% serum containing Fengbaisan and EX527.

### Quantitative real-time PCR (qRT-PCR)

QRT-PCR was used to measure the expression intensity of different genes. Total RNA was extracted from lung tissues and cells using TRIzol reagent (Invitrogen, Carlsbad, CA, USA). The purity and quantity of RNA was detected using NanoDrop 2000 spectrophotometer (Thermo Fisher Scientific, Waltham, MA, USA). The RNA was reversely transcribed to complementary DNA using PrimeScript™ RT Reagent Kit (Takara, Tokyo, Japan). QRT-PCR was carried out using SYBR Green PCR Mix Kit (Takara) according to the instruction. The results were analysed using the ΔΔCT (cycle threshold) method for quantification.

### Western blot (WB)

Total protein was extracted from lung tissues or cells using Tissue or Cell Total Protein Extraction Kit (Sangon Biotech). Equivalent protein from different samples was separated by protein electrophoresis, following by transformation onto PVDF membranes (Merck Millipore, Billerica, MA, USA). The membranes were incubated with the anti-rabbit SIRT1, TIMP-1, MMP-9, CHOP, GRP78, caspase-12 or caspase-3 antibodies (1:1000 dilution, Proteintech, Wuhan, China) at 4 °C overnight after immersed into blocking buffer. After the membranes were washed with TBST for several times, goat anti-mouse IgG antibody (1:5000, Proteintech) labelled with horseradish peroxidase were incubated with the membranes as a secondary antibody. Anti-mouse β-actin antibody (1:5000, Proteintech) was used as a reference protein for normalization. The grey levels of the protein bands were examined by Image J software.

### Flow cytometry

The cells were collected by centrifuging for 5 min at the speed of 500 *g*, 4 °C. The cells were washed with pre-cooling PBS for 2 times. Cells were then resuspended in the Annexin V Binding buffer. The cell suspension was dyed with Annexin V-FITC and PI and plunged into darkness at room temperature for 15 min. Then, the cell suspension was mixed with Annexin V Binding buffer and put on ice. The apoptosis rate of cells was determined by flow cytometry in an hour. The assay was performed according to the instruction of Annexin V-FITC/PI Cell Apoptosis Detection Kit (TransGen Biotech, Beijing, China).

### Enzyme linked immunosorbent assay (ELISA)

The content of IL-1β, TNF-α and IL-8 in BALF was assessed using Rat IL-1β ELISA Kit (TW-reagent, Shanghai, China), Rat TNF-α ELISA Kit (TW-reagent) and Rat IL-8 ELISA Kit (TW-reagent). The assay was performed according to the manufacturer’s instructions. The optical density values of samples were detected at 450 nm wavelength using enzyme-labelled instrument (Thermo Fisher Scientific).

### Detection of total inflammatory cells and neutrophils

The total number of inflammatory cells in BALF was counted using a haemocytometer. BALF from rats was made into smears. The number and percentage of neutrophils in BALF was counted using Wright-Giemsa stain (Solarbio, Beijing, China) following the manufacturer’s protocol.

### Hematoxylin-eosin (HE) staining or TdT-mediated dUTP nick-end labelling (TUNEL) assay

Fresh lung tissues were fixed in 4% paraformaldehyde and embedded in paraffin. Five-micron sections were obtained after deparaffin and rehydration. Then, sections were stained using HE staining kit (Solarbio) to observe the pathological changes of lung tissues. To assess the levels of apoptosis of lung tissues, paraffin sections were stained using TUNEL Apoptosis Assay Kit following the manufacturer’s protocol. The stained sections were then observed under the Nikon microscope (Nikon, Tokyo, Japan).

### Statistical analysis

All values were exhibited as mean ± standard deviation and analysed by SPSS 22.0 statistical software (IBM, Armonk, NY, USA). For comparison of two groups, a two-tailed Student’s *t*-test was used. Comparison of multiple groups was made using a one- or two-way ANOVA. Difference was considered statistically significant at *p* < 0.05.

## Results

### Fengbaisan treatment facilitates SIRT1 expression in lung tissues of COPD rats

**Figure 1.**
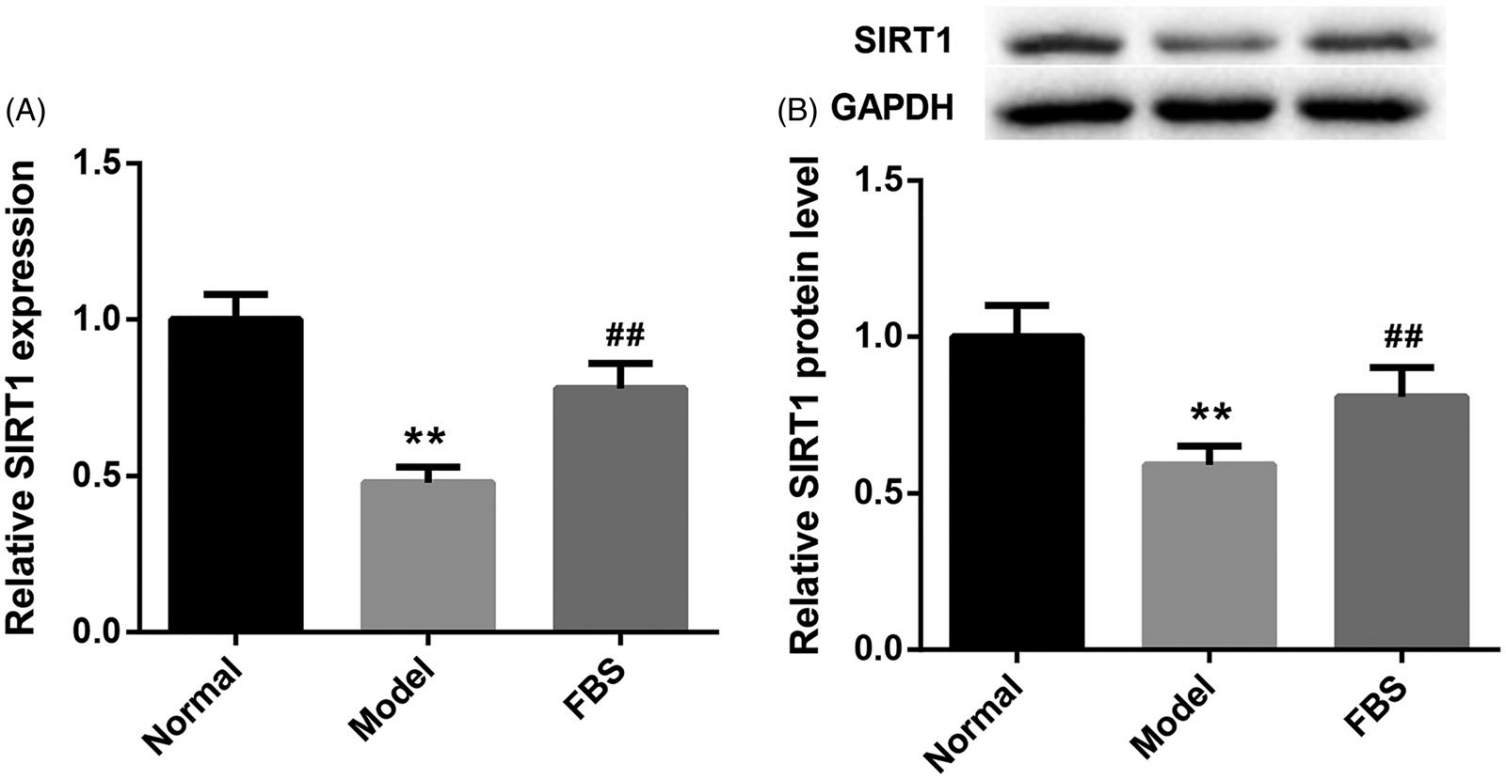
Fengbaisan treatment facilitates SIRT1 expression in lung tissues of COPD rats. Rats were challenged with CSE and LPS for 4 weeks with or without Fengbaisan. (A) QRT-PCR was performed to validate SIRT1 gene expression in lung tissues of rats. (B) WB was performed to assess SIRT1 protein expression in lung tissues of rats. (**p < 0.01, vs. Normal; ^##^*p* < 0.01, vs. Model).

To investigate the involvement of SIRT1 in COPD, we analysed its expression in lung tissues of normal and COPD rats by qRT-PCR. Compared with normal rats, the gene and protein expression of SIRT1 was significantly downregulated in lung tissues of COPD rats. Fengbaisan treatment led to a boost in the gene and protein expression of SIRT1 in lung tissues of COPD rats (Figure 1(A,B)). This indicates that Fengbaisan treatment facilitates SIRT1 expression in lung tissues of COPD rats.

### Fengbaisan treatment improves the lung function of COPD via SIRT1 pathway

To investigate whether Fengbaisan had a protective effect on COPD, we examined the lung function of COPD rats, including FEV0.3/FVC, Cdyn and RI. Compared with normal rats, FEV0.3/FVC (57.65%) and Cdyn (0.16 cm H_2_O/mL/s) were severely decreased, whereas RI (0.67 mL/cm H_2_O) was obviously enhanced in Model group. However, Fengbaisan treatment significantly promoted FEV0.3/FVC (74.28%) and Cdyn (0.28 cm H_2_O/mL/s), and inhibited RI (0.48 mL/cm H_2_O) as compared with the Model group. In addition, the influence of Fengbaisan on lung function was abolished by EX527 (SIRT1 inhibitor) treatment (Table 1). The results show that Fengbaisan treatment improves the lung function of COPD via SIRT1 pathway.

**Table 1.** Comparison of FEV0.3/FVC, Cdyn and RI in rats.

Group	n	FEV0.3/FVC (%)	Cdyn (cmH_2_O/ml/s)	RI (ml/cmH_2_O)
Normal	8	82.39 ± 5.44	0.35 ± 0.03	0.35 ± 0.04
Model	8	57.65 ± 3.81^a^	0.16 ± 0.02^a^	0.67 ± 0.08^a^
FBS	8	74.28 ± 5.33^b^	0.28 ± 0.04^b^	0.48 ± 0.04^b^
FBS + DMSO	8	75.93 ± 5.64	0.27 ± 0.02	0.5 ± 0.03
FBS + EX527	8	63.11 ± 4.08^c^	0.19 ± 0.02^c^	0.59 ± 0.05^c^

^a^*p* < 0.05, versus Normal; ^b^*p* < 0.05, versus Model; ^c^*p* < 0.05, versus FBS + DMSO.

### Fengbaisan treatment represses ERS and apoptosis by upregulating SIRT1 expression in A549 cells

**Figure 2.**
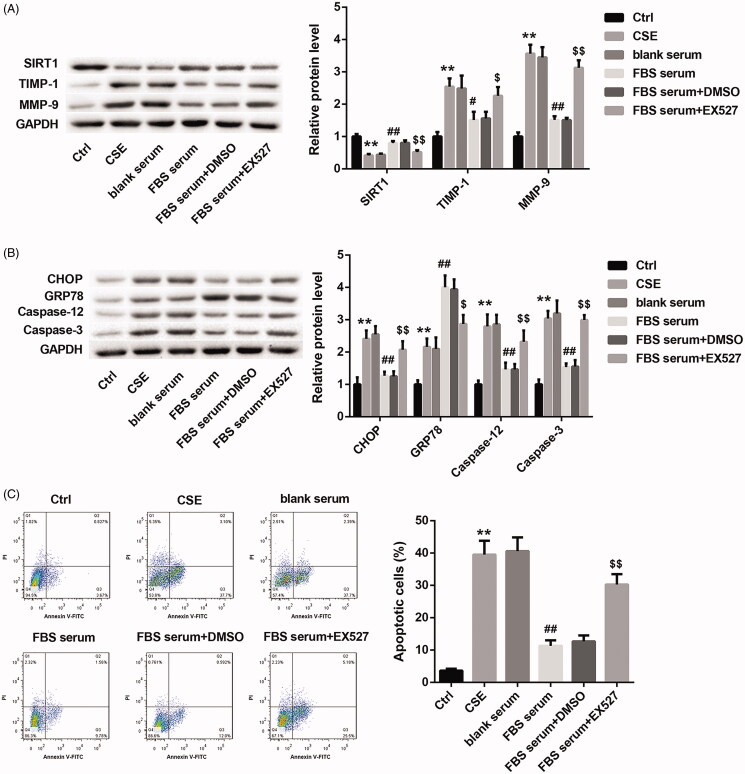
Fengbaisan treatment represses ERS and apoptosis by upregulating SIRT1 expression in A549 cells *in vitro*. A549 cells were treated with 10% CSE. Then, the CSE-induced A549 cells were treated with blank serum, serum containing Fengbaisan, serum containing Fengbaisan and DMSO or serum containing Fengbaisan and EX527. A549 cells did not receive special treatment as control. (A, B) WB was performed to assess the expression of SIRT1, TIMP-1, MMP-9, CHOP, GRP78, caspase-12 and caspase-3 in the A549 cells. (C) Flow cytometry was performed to examine the levels of apoptosis in the A549 cells. (**p < 0.01, vs. Ctrl; ^#^*p* < 0.05, vs. blank serum; ^##^*p* < 0.01, vs. blank serum; ^$^*p* < 0.05, vs. FBS serum + DMSO; ^$$^*p* < 0.01, vs. FBS serum + DMSO).

In order to define the role of Fengbaisan treatment in A549 cells, CSE-induced A549 cells were treated with the serum-derived from COPD rats of each group. As shown in Figure 2(A), the CSE group exhibits a decrease of SIRT1 expression and an increase of TIMP-1 and MMP-9 expression as compared with the Ctrl group. Fengbaisan-contained serum treatment caused a boost of SIRT1 expression and a decrease of TIMP-1 and MMP-9 expression in CSE-induced A549 cells, which was effectively abolished by EX527 treatment (Figure 2(A)). Then, we investigated the expression of CHOP, GRP78, caspase-12 and caspase-3 in the A549 cells by WB. The expression of CHOP, GRP78, caspase-12 and caspase-3 in CSE group was significantly higher than that in Ctrl group. FBS serum group exhibited a pronounced decrease in the expression of CHOP, caspase-12 and caspase-3, and caused an obvious increase of GRP78 expression as compared with the blank serum group. The influence conferred by Fengbaisan was rescued by EX527 treatment (Figure 2(B)). In addition, flow cytometry was performed to examine the levels of apoptosis in A549 cells. CSE group exhibited a boost in apoptosis with respect to Ctrl group. FBS-contained serum treatment led to a decrease in apoptosis, which was effectively abolished by EX527 treatment (Figure 2(C)). These data taken together imply that Fengbaisan treatment facilitates SIRT1 expression and represses ERS and apoptosis in A549 cells *in vitro*.

### Fengbaisan treatment suppresses airway inflammation via SIRT1 pathway in lung tissues of COPD rats

**Figure 3.**
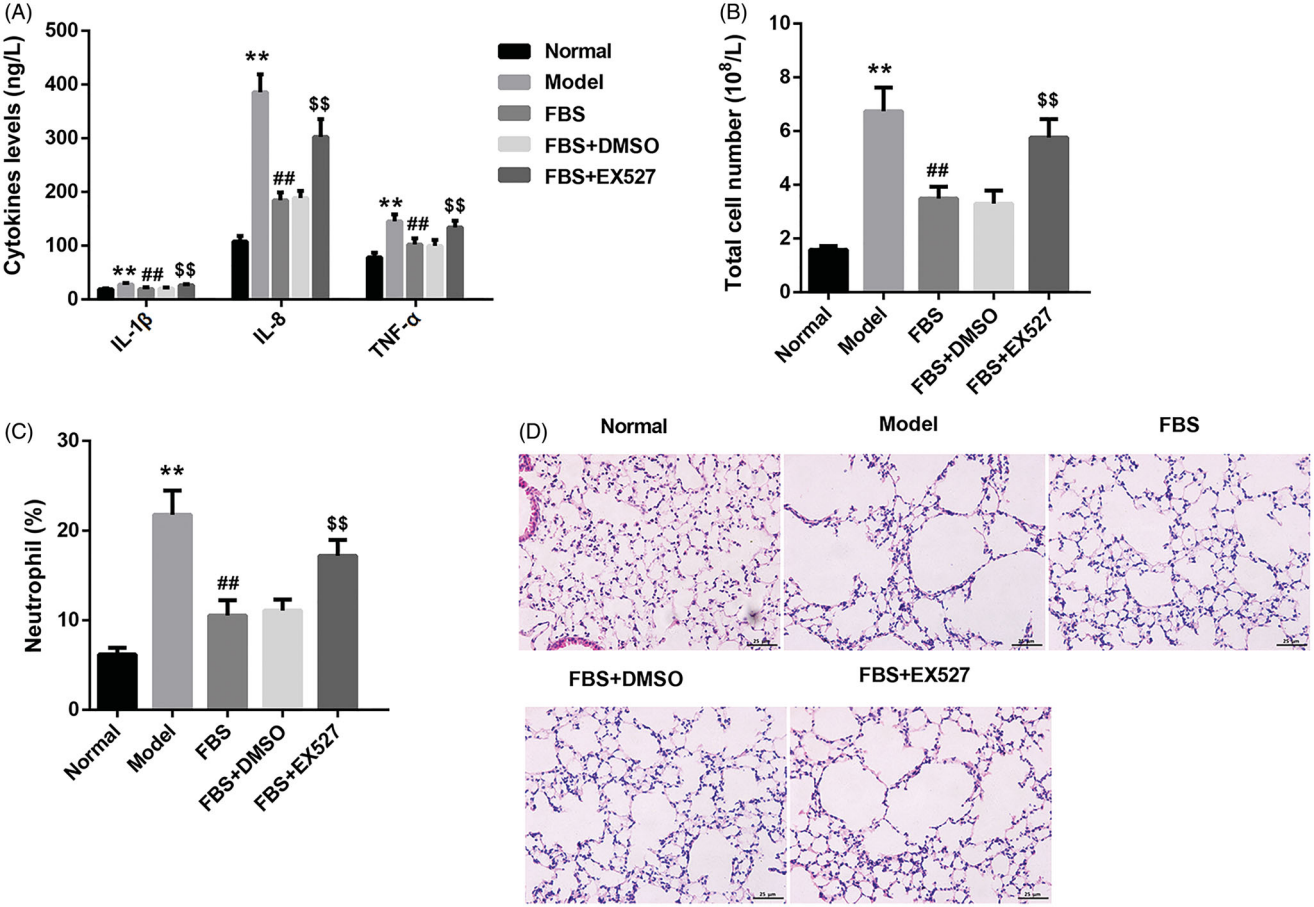
Fengbaisan treatment suppresses airway inflammation via SIRT1 pathway in lung tissues of COPD rats. Rats were challenged with CSE and LPS to construct rat model of COPD. COPD rats were treated with FBS, FBS + DMSO or FBS + EX527. Normal rats did not receive special treatment as control. (A) ELISA was performed to validate the levels of IL-1β, TNF-α and IL-8 in BALF of rats. (B) The total number of inflammatory cells in BALF of rats was counted using haemocytometer. (C) The percentage of neutrophils in BALF of rats was evaluated by Wright-Giemsa staining. (D) The pathological changes of lung tissues of rats were observed by HE staining. (**p < 0.01, vs. Normal; ^##^*p* < 0.01, vs. Model; ^$$^*p* < 0.01, vs. FBS + DMSO).

The airway inflammation is closely associated with the development of COPD (Guo et al. 2019). To verify the effect of Fengbaisan on airway inflammation in COPD, we examined the levels of IL-1β, TNF-α and IL-8, total number of inflammatory cells and the percentage of neutrophils in BALF of rats. The levels of IL-1β, TNF-α and IL-8 in BALF were notably enhanced in Model group with respect to Normal group. Compared with Model group, the levels of IL-1β, TNF-α and IL-8 in BALF were severely suppressed in FBS group. And the influence conferred by Fengbaisan was abolished by EX527 treatment (Figure 3(A)). Moreover, the total number of inflammatory cells and the percentage of neutrophils in BALF were enhanced in Model group as compared with Normal group. Fengbaisan treatment caused a decrease of the total number of inflammatory cells and the percentage of neutrophils in BALF. Instead, the inhibitory effect of Fengbaisan treatment on the total number of inflammatory cells and the percentage of neutrophils in BALF was rescued by EX527 treatment (Figure 3(B,C)). In addition, we observed the pathological changes of lung tissues in COPD rats by HE staining. Compared with Normal group, Model group exhibited an increase of lung damage. Fengbaisan treatment attenuated the levels of lung damage in COPD rats. And the influence conferred by Fengbaisan treatment was abolished by EX527 treatment (Figure 3(D)). These data indicate that Fengbaisan treatment suppresses airway inflammation via SIRT1 pathway in lung tissues of COPD rats.

### Fengbaisan treatment suppresses ERS and apoptosis by upregulating SIRT1 expression in lung tissues of COPD rats

**Figure 4.**
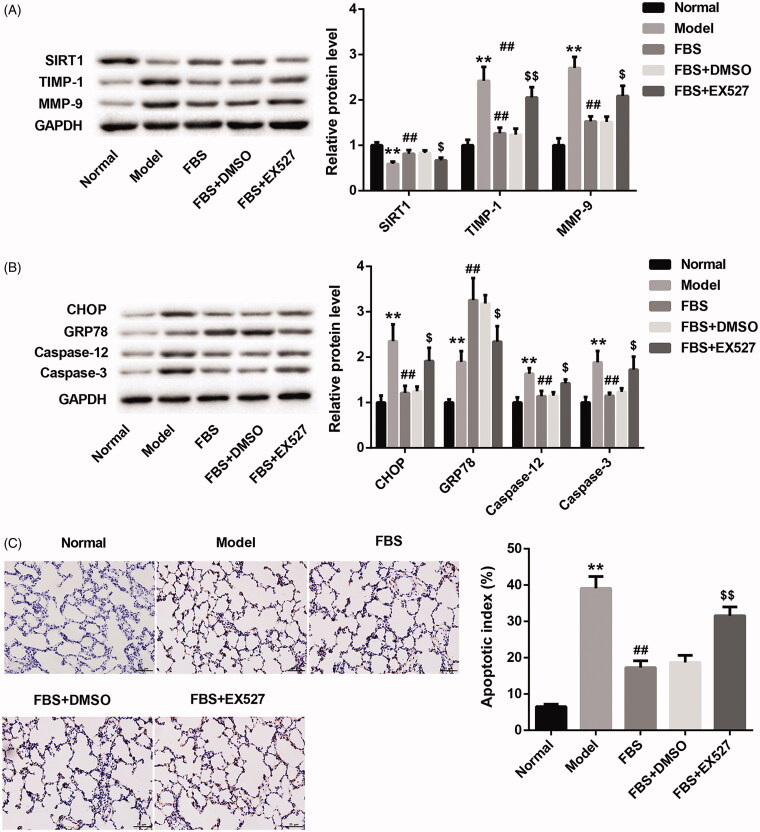
Fengbaisan treatment suppresses ERS and apoptosis by upregulating SIRT1 expression in lung tissues of COPD rats. Rats were challenged with CSE and LPS to construct rat model of COPD. COPD rats were treated with FBS, FBS + DMSO or FBS + EX527. Normal rats did not receive special treatment as control. (A, B) WB was performed to assess the expression of SIRT1, TIMP-1, MMP-9, CHOP, GRP78, caspase-12 and caspase-3 in lung tissues of COPD rats. (C) TUNEL staining was performed to examine the levels of apoptosis in lung tissues of COPD rats. (**p < 0.01, vs. Normal; ^##^*p* < 0.01, vs. Model; ^$$^*p* < 0.01, vs. FBS + DMSO).

To verify whether Fengbaisan could improve COPD via SIRT1 pathway, we measured the expression of SIRT1, TIMP-1 and MMP-9 in COPD rats. As shown in Figure 4(A), the Model group exhibits a decrease of SIRT1 expression and an increase of TIMP-1 and MMP-9 expression as compared with the Normal group. Compared with Model group, Fengbaisan treatment caused a boost of SIRT1 expression and a decrease of TIMP-1 and MMP-9 expression in FBS group, which was effectively abolished by EX527 treatment (Figure 4(A)). Then, we investigated the expression of CHOP, GRP78, caspase-12 and caspase-3 in COPD rats by WB. The expression of CHOP, GRP78, caspase-12 and caspase-3 in Model group was significantly higher than that in Normal group. FBS group exhibited a pronounced decrease in the expression of CHOP, caspase-12 and caspase-3, and caused an obvious increase of GRP78 expression with respect to Model group. The influence conferred by Fengbaisan was abolished by EX527 treatment (Figure 4(B)). In addition, flow cytometry was performed to examine the apoptosis in COPD rats. Model group displayed an increase in apoptosis with respect to Normal group. Fengbaisan treatment led to a decrease in apoptosis. The inhibiting effect of Fengbaisan on apoptosis was rescued by EX527 treatment (Figure 4(C)). These data taken together suggest that Fengbaisan treatment suppresses ERS and apoptosis by upregulating SIRT1 expression in lung tissues of COPD rats.

## Discussion

Recently, investigators have examined the role of SIRT1 in COPD. SIRT1 upregulation reduces oxidative stress and modulate epithelial-mesenchymal transition by activating TGF-β1/Smad3 signalling pathway, thereby ameliorating cigarette smoke-induced airway remodelling and COPD (Guan et al. 2020). Curcumin ameliorates COPD by promoting autophagy and inhibiting ERS through regulation of SIRT1 in COPD rats (Tang and Ling 2019). Melatonin inhibits cigarette smoke-induced inflammatory mediators by upregulating SIRT1 expression, thereby improving COPD (Shin et al. 2020). These data imply that SIRT1 has a pivotal role in the occurrence and development of COPD. In our study, SIRT1 expression was notably downregulated in COPD rats, and Fengbaisan treatment led to an increase of SIRT1 expression in COPD rats, suggesting that SIRT1 was associated with COPD.

Recent evidence suggests that SIRT1 plays a key role in the control of COPD by regulating ERS and autophagy-mediated apoptosis (He et al. 2019a). Hydrogen treatment reduces the expression of CHOP, GRP78 and alleviates hyperoxic acute lung injury related-ERS in rats (Sun et al. 2017). SIRT1 regulates the imbalance of TIMP-1/MMP-9 in the lungs of COPD patients to slow the occurrence and development of COPD and emphysema (Yao et al. 2013). Our data showed that SIRT1 was obviously decreased in CSE-induced A549 cells. The expression of TIMP-1 and MMP-9 was upregulated in CSE-induced A549 cells. Numerous studies have demonstrated that TIMP-1 and MMP-9 are highly expressed in lung tissues of COPD patients, and the imbalance of TIMP-1/MMP-9 is closely associated with the occurrence of emphysema and COPD (Higashimoto et al. 2005; Calikoğlu et al. 2006). And the expression of ERS-related genes (CHOP and GRP78) and apoptotic gene (caspase-3 and caspase-12) expression was increased in CSE-induced A549 cells, suggesting that CSE induced ERS and apoptosis in A549 cells. The levels of apoptosis in CSE-induced A549 cells were increased. In addition, Fengbaisan treatment promoted SIRT1 and GRP78 expression, and caused a decrease of the expression of TIMP-1, MMP-9, CHOP, caspase-3 and caspase-12 and the levels of apoptosis in A549 cells. However, this influence conferred by Fengbaisan treatment was abolished by EX527 treatment in A549 cells. These data taken together reveal that Fengbaisan treatment represses ERS and apoptosis by upregulating SIRT1 expression in A549 cells *in vitro*.

In COPD rats, the changes of FEV0.3/FVC, Cdyn and RI showed that the lung function was significantly damaged. And the lung damage was rescued by Fengbaisan treatment. EX527 (SIRT1 inhibitor) treatment inhibited the effect of Fengbaisan on lung function, indicating that Fengbaisan treatment improves the lung function of COPD via SIRT1 pathway. Moreover, the levels of inflammatory factors (IL-1β, TNF-α and IL-8), the total number of inflammatory cells and the percentage of neutrophils in BALF were notably enhanced, implying that the degree of inflammation was increased in lung of COPD rats. The inflammatory reaction was effectively repressed by Fengbaisan treatment. The effect of Fengbaisan on inflammatory reaction was rescued by EX527 treatment. Therefore, Fengbaisan treatment suppresses airway inflammation via SIRT1 pathway in lung tissues of COPD rats. In addition, Fengbaisan treatment promoted SIRT1 expression, and inhibited the expression of TIMP-1/MMP-9, CHOP, caspase-12 and caspase-3 in COPD rats. Fengbaisan caused a decrease in apoptosis of COPD rats. However, the influence on TIMP-1/MMP-9, ERS and apoptosis conferred by Fengbaisan was abolished by EX527 treatment. Thus, these data taken together imply that Fengbaisan treatment suppresses ERS and apoptosis by upregulating SIRT1 expression in lung tissues of COPD rats.

## Conclusions

Our studies demonstrate that Fengbaisan exerts a protective effect against COPD. The protective effect of Fengbaisan is attributed to the inhibition of ERS and inflammation reaction by upregulating SIRT1 expression in lung tissues of COPD rats. Therefore, our data provide evidence that Fengbaisan could be a promising therapeutic target drug for COPD treatment.
